# TGR5 Activation Modulates an Inhibitory Effect on Liver Fibrosis Development Mediated by Anagliptin in Diabetic Rats

**DOI:** 10.3390/cells8101153

**Published:** 2019-09-26

**Authors:** Daisuke Kaya, Kosuke Kaji, Yuki Tsuji, Satoko Yamashita, Koh Kitagawa, Takahiro Ozutsumi, Yukihisa Fujinaga, Hiroaki Takaya, Hideto Kawaratani, Kei Moriya, Tadashi Namisaki, Takemi Akahane, Hitoshi Yoshiji

**Affiliations:** 1 Third Department of Internal Medicine, Nara Medical University, Kashihara, Nara 634-8521, Japan; kayad@naramed-u.ac.jp (D.K.); tsujih@naramed-u.ac.jp (Y.T.); kitagawa@naramed-u.ac.jp (K.K.); ozutaka@naramed-u.ac.jp (T.O.); fujinaga@naramed-u.ac.jp (Y.F.); htky@naramed-u.ac.jp (H.T.); kawara@naramed-u.ac.jp (H.K.); moriyak@naramed-u.ac.jp (K.M.); tadashin@naramed-u.ac.jp (T.N.); stakemi@naramed-u.ac.jp (T.A.); yoshijih@naramed-u.ac.jp (H.Y.); 2 Sanwa Kagaku Kenkyusho, Co., Ltd., Nagoya, Aichi 461-8631, Japan; s_yamashita@skk-net.com

**Keywords:** dipeptidyl peptidase-4 inhibitors, hepatic stellate cells, oleanolic acid, Takeda G protein-coupled receptor 5

## Abstract

Hyperglycemia and hyperinsulinemia activate the proliferative potential of hepatic stellate cells (HSCs) and promote hepatic fibrosis. Dipeptidyl peptidase-4 (DPP-4) inhibitors, antidiabetic agents, reportedly inhibit the HSC proliferation. Additionally, Takeda G protein-coupled receptor 5 (TGR5) agonists induce the systemic release of glucagon-like peptides from intestinal L cells, which maintains glycemic homeostasis. This study assessed the combined effect of TGR5 agonist and DPP-4 inhibitor on diabetes-based liver fibrosis development. Male diabetic rats received intraperitoneal injection of porcine serum (PS) to induce liver fibrosis, and they were orally administered the following agents: oleanolic acid (OA) as a TGR5 agonist, anagliptin (ANA) as a DPP-4 inhibitor, and a combination of both agents. Treatment with OA or ANA significantly improved glycemic status and attenuated intrahepatic steatosis and lipid peroxidation in diabetic rats. PS-induced liver fibrosis development was also drastically suppressed by treatment with either agent, and the combination of both reciprocally enhanced the antifibrotic effect. Fecal microbiome demonstrated that both agents inhibited the increase in the Firmicutes/Bacteroidetes ratio, an indicator of dysbiosis related to metabolic syndromes. Furthermore, ANA directly inhibited in vitro HSC proliferative and profibrogenic activities. Collectively, TGR5 agonist and DPP-4 inhibitor appears to be a novel strategy against liver fibrosis under diabetic conditions.

## 1. Introduction

Liver fibrosis is a common feature of chronic liver injuries caused by a variety of etiologies (e.g., hepatitis B, hepatitis C, autoimmune disorders, alcohol abuse, and non-alcoholic fatty liver disease (NAFLD)) [1,2,3]. Pathologically, liver fibrosis is characterized by hepatic stellate cell (HSC) activation and excessive accumulation in the extracellular matrix. The progression of liver fibrosis is often influenced by various pathological conditions. Among others, the diabetic conditions type 2 diabetes mellitus (T2DM) and insulin resistance (IR) are crucial to aggravate fibrogenesis [4,5]. Several epidemiological studies revealed that IR represents an advanced fibrosis risk factor in patients with chronic hepatitis C [6,7]. Of note, T2DM and IR are known risk factors for progressive fibrosis in NAFLD patients. Remarkably, in adult patients with non-alcoholic steatohepatitis (NASH), T2DM has been reported to affect the presence of progressive liver fibrosis as well as the progression to advanced fibrosis [8]. In fact, an earlier study found that either high glucose or insulin increased the production and expression of collagen genes in the activated hepatic stellate cells (HSCs) playing a central role in liver fibrosis as the major source of fibrogenic cells [4,5]. Moreover, in a previous report from our group, we elucidated that the IR status, i.e., co-existence of high glucose and insulin, directly augments activated HSCs’ in vitro proliferative potential [9].

Importantly, a recent longitudinal study in NASH patients demonstrated that the fibrosis stage is uniquely associated with long-term overall mortality and liver-related events [10]. This evidence emphasizes the importance of curative therapies aimed at improving NASH-based fibrosis development. However, to date, antifibrotic agents have not been clinically established. Therefore, an alternative strategy, until new drugs become entrenched, may be to identify available compounds showing also antifibrotic activity. We have recently demonstrated that a dipeptidyl peptidase-4 (DPP-4) inhibitor successfully attenuated liver fibrosis. Specifically, this agent acted by inhibiting platelet-derived growth factor-BB-induced phosphorylation of Smad2/3, p38 MAPK, and ERK1/2 in activated HSCs [11]. However, recent randomized controlled trials suggested that DPP-4 inhibitor alone does not improve liver fibrosis in NAFLD patients [12,13]. Specifically, it has been shown combined therapy is required to enhance the compounds’ antifibrotic effects.

The G protein-coupled bile acid receptor 1, known as Takeda G protein-coupled receptor (TGR5), is a cell-surface receptor extensively expressed in various human tissues (e.g., stomach, liver, lung, skeletal muscle, spleen, adipose tissue, and intestine) [14]. TGR5 can be activated by bile acids and it plays key roles in cell signaling pathways (i.e., extracellular signal-regulated kinases (ERK), nuclear factor-κB, and AKT [15,16,17]. It has been shown that TGR5’s activation provides beneficial effects on various metabolic diseases such as obesity, dyslipidemia, and T2DM [18]. Of note, TGR5 agonists stimulate the systemic release of glucagon-like peptide (GLP)-1 and -2 and peptide YY in intestinal L cells, a type of enteroendocrine cells [19]. GLP-1 is known to be an incretin that exerts insulinotropic activities in the pancreas by regulating glucose homeostasis. GLP-1 analogs have been established as antidiabetic agents [20,21]. Moreover, basic studies on NASH rodent models have revealed that GLP-1 could attenuate hepatic steatosis, inflammation, and fibrosis [22,23,24]. Additionally, a clinical evidence has demonstrated that liraglutide, a GLP-1 analog, improves histological features in NASH patients [25].

Based on the biological interactions highlighted here between TGR5 and GLP-1, TGR5 has become an enticing potential target for NASH therapeutics. Of note, INT-777, a TGR5 agonist, has been shown to improve insulin sensitivity. Specifically, this occurs through increased GLP-1 release in the intestine, resulting in the reduction of hepatic steatosis with lowered liver enzyme levels in diet-induced obese mice [26]. As a consequence, TGR5 agonists have attracted attention as therapeutic candidates for diabetes-based liver fibrosis including NASH, particularly in combination with a DPP-4 inhibitor.

This study aims to evaluate the joint effect of the DPP-4 inhibitor anagliptin (ANA) and the TGR5 agonist oleanolic acid (OA) on liver fibrogenesis, and analyze the therapeutic mechanisms using a rat model with congenital diabetes.

## 2. Materials and Methods

### 2.1. Animals and Reagents

Male Otsuka Long–Evans Tokushima fatty (OLETF) rats and littermate Long–Evans Tokushima Otsuka (LETO) rats were purchased from Japan SLC, Inc. (Hamamatsu, Japan). ANA, a DPP-4 inhibitor, was supplied by Sanwa Kagaku Kenkyusho CO., LTD (Nagoya, Japan). OA (Tokyo Chemical Industry CO., LTD., Tokyo, Japan) was used as a TGR5 agonist.

### 2.2. Animal Treatment

Rats were housed in stainless steel mesh cages under controlled conditions (temperature: 23 °C ± 3 °C; relative humidity: 50% ± 20%; 10–15 air changes/h; illumination: 12 h/d). Ad libitum access to tap water throughout the study period was given to the rats. In order to induce liver fibrosis, 16-week-old OLETF rats underwent an intraperitoneal injection of 1 mL/kg porcine serum (PS) (Cosmo Bio, Tokyo, Japan) twice a week for 8 weeks. Of note, an equal amount of saline was injected in the negative control group. The rats were divided into the following five treatment groups (*n* = 10 each): negative control, PS injection with vehicle, OA, ANA, and combined agents. During the same period as PS administration, the OLETF rats’ diet contained a mixture of 100 mg/kg/day of OA and/or 45 mg/kg/day of ANA (CLEA Japan, Inc., Osaka, Japan). At the end of experiments, all of the 24-week-old rats underwent the following procedures: anesthesia, opening of their abdominal cavities, blood collection via aortic puncture, gathering of feces from the terminal ileum for microbiome analysis, and harvesting of livers for histological evaluation. Additionally, from the negative control groups, other organs were harvested in order to evaluate the tissue distribution of TGR5 expression. Routine laboratory methods were used to measure serum biological markers. All animal procedures were performed based on the recommendations of the Guide for Care and Use of Laboratory Animals (National Research Council). The animal facility committee of Nara Medical University (Authorization number: 12052) approved this study.

### 2.3. Cell Culture

The human enteroendocrine L cell line NCI-H716 (American Type Culture Collection, Manassas, VA, USA) was cultured as described previously [27]. Briefly, the cells were initially grown in low-glucose RPMI-1640 (Nacalai Tesque, Kyoto, Japan) with 10% fetal bovine serum (FBS) (Biosera, Kansas City, MO, USA) at 37 °C and in the presence of 5% CO_2_. Subsequently, in order to generate mature endocrine cells, they were maintained in Matrigel (Corning, Bedford, MA, USA) with high glucose Dulbecco’s modified Eagle’s medium (DMEM) (Nacalai Tesque) and 10% FBS for 2 days. Furthermore, 100 U/mL penicillin and 100 mg/mL streptomycin were added to the culture media at a ratio of 1:1000.

LX-2 human stellate cells and HSC-T6 rat stellate cells were purchased from Merck KGaA (Darmstadt, Germany). Both lines were maintained as monolayer cultures in DMEM with 10% FBS and 1% penicillin/streptomycin in an incubator at 37 °C and 5% CO_2_. For each assay, cells were pre-incubated for 6 h with 5 ng/mL of human and rat transforming growth factor-β1 (TGF-β1) (Sigma-Aldrich, St. Louis, MO, USA).

### 2.4. Cell Proliferation Assay

In order to evaluate the direct effect of ANA and OA on human and rat HSC lines, cell proliferation following treatment with or without both reagents was compared. LX-2 and HSC-T6 cells were seeded on uncoated plastic dishes at a density of 5 × 10^4^ cells/mL. Following an overnight culture, the cells were treated with different concentrations of ANA (0–1000 μM) or OA (0–75 μM) for 24 h after pre-treatment with TGF-β1. Cell proliferation was measured with the WST-1 assay (Takara Bio Inc., Kusatsu, Japan) according to the manufacturer’s manual.

### 2.5. RNA Extraction and Quantitative Real-Time-PCR

Total RNA was extracted from frozen liver tissues, NCI-H716, LX-2, and HSC-T6 cells using the RNeasy mini kit (QIAGEN, Tokyo, Japan), as per manufacturer’s instructions. Total RNA (2 μg) from each sample was subsequently reverse transcribed into complementary DNA (cDNA). To this end, a high-capacity RNA-to-cDNA kit (Applied Biosystems Inc., Foster City, CA, USA) was used, following the manufacturer’s instructions. cDNA quantitative real-time PCR was performed using gene-specific primer pairs (Supplementary Materials Table S1) and Step One Real-Time PCR (Applied Biosystems Inc.). Glyceraldehyde-3-phosphate dehydrogenase was used as an internal control to measure relative gene expression. The relative amount of target mRNA per cycle was determined by applying a threshold cycle to the standard curve. Human skeletal muscle and spleen tissues (Takara Bio Inc.) were employed as controls for mRNA levels of human *GPBAR1*.

### 2.6. Protein Extraction and Western Blotting

Whole cell lysates were prepared from 10^6^ cultured NCI-H716, LX-2, and HSC-T6 cells. LX-2 and HSC-T6 cells were incubated with OA or ANA for 12 h after pretreatment with TGF-β1. For this purpose, T-PER Tissue Protein Extraction Reagent supplemented with proteinase and phosphatase inhibitors (all Thermo Scientific, Rockford, IL, USA) was used. Fifty micrograms of whole cell lysates were separated by SDS-PAGE. Subsequently, they were transferred to a PVDF membrane that was then blocked with 5% bovine serum albumin in Tris-buffered saline + Tween-20 for 1 h. Thereafter, each membrane was incubated overnight with the following antibodies against: GPCR-TGR5 (abcam), extracellular signal-regulated kinase (ERK 1/2), phospho-ERK1/2 (Thr202/Tyr204), Smad2, phospho-Smad2 (Ser465/467), Smad3, phospho-Smad3 (Ser423/425), and β-Actin (Cell Signaling Technology). The membranes were then washed and incubated with HRP-linked F(ab)2 fragment (GE Healthcare Life Sciences, Piscataway, NJ, USA; 1:5000 dilution). Finally, Clarity Western ECL Substrate (BIORAD, Hercules, CA, USA) was used to develop each membrane.

### 2.7. Intrahepatic Thiobarbituric Acid Reactive Substances (TBARS) Measurement

Whole cell lysates were prepared from 200 mg of frozen liver tissue, as described above. Intrahepatic TBARS were assessed by measuring the hepatic content of malondialdehyde (MDA). To this end, a TBARS Assay Kit (Cayman Chemical, Ann Arbor, MI, USA) was used.

### 2.8. Measurement of Cyclic AMP (cAMP) and GLP-1 Levels

Mature enteroendocrine NCI-H716 cells were cultured in high glucose DMEM, which included 0.2% BSA (Nacalai Tesque). The cells were treated with 0.1% ethanol as solvent control or OA (1, 5, and 10 μM). Following a 2 h treatment, the cells were collected and lysed with T-PER. The levels of cAMP in the cell lysate were determined by using a cAMP ELISA kit (Cayman Chemical) and rectified with cell protein concentration. In order to investigate the effects of OA on GLP-1 secretion, mature NCI-H716 cells were incubated in KRB buffer (Sigma-Aldrich) and 0.2% FBS. The cells were treated with 0.1% ethanol and increasing concentrations of OA (1, 5, and 10 μM) for 2 h. GLP-1 levels in the cellular supernatant supplemented with 50 μg/mL PMSF (Nacalai Tesque) were then measured with a GLP-1 ELISA assay (Mercodia, Uppsala, Sweden). Furthermore, the serum levels of rat GLP-1 were measured by GLP-1 ELISA Kit Wako, High Sensitive (Fujifilm Wako Pure Chemical Co., Osaka, Japan), according to the manufacturer’s instruction.

### 2.9. Statistical Analyses

Data were subjected to Student’s *t*-test or one-way analysis of variance followed by Bonferroni’s multiple-comparison test, as appropriate. Bartlett’s test was used to determine the homology of variance. Correlations were calculated with the Spearman’s rank test. A two-tailed *p*-value ≤ 0.05 was considered as statistically significant. Analyses were performed with EZR (Saitama Medical Center, Jichi Medical University). The latter is a graphical user interface for R (The R Foundation for Statistical Computing, version 2.13.0). Specifically, EZR represents a modified version of the R commander (version 1.6-3). Of note, it includes statistical functions frequently used in biostatistics [28]. Additional methods can be found online in the Supplementary Materials.

## 3. Results

### 3.1. Oleanolic Acid Stimulates GLP-1 Synthesis and Secretion via TGR5 Activation in Human Intestinal Cells

**Figure 1:**
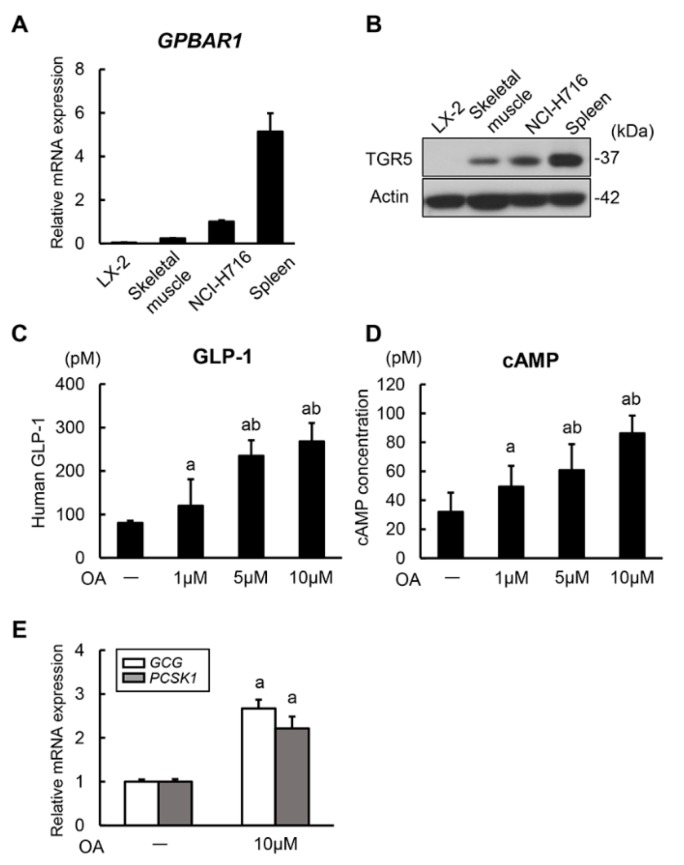
GLP-1 synthesis and secretion in human intestinal cells. (**A**) Relative mRNA expression levels of human *GPBAR1*. Quantitative values are indicated as ratios to the values of NCI-H716. Human skeletal muscle and spleen tissues were used as controls. (**B**) Western blots of whole cell lysates for the expression of TGR5. Actin was used as internal control for western blotting. (**C**) Measurement of human GLP-1 concentrations in NCI-H716 cell-cultured media. NCI-H716 cells were cultured with different concentrations of oleanolic acid (OA) for 2 h. (**D**) Measurement of human intracellular cAMP concentrations in NCI-H716 cells. NCI-H716 cells were cultured with different concentrations of oleanolic acid for 2 h. (**E**) Relative mRNA expression levels of human *GCG* and *PCSK1* in NCI-H716 cells treated without and with oleanolic acid (10 μM) for 2 h. Quantitative values are indicated as ratios to the values of OA (–)-group. Relative mRNA expression levels were measured by quantitative RT-PCR (qRT-PCR). *GAPDH* was used as internal control for qRT-PCR (**A**,**D**). Data are mean ± SEM (*n* = 8). ^a^, *P* ≤ 0.05 compared with OA (–)-group; ^b^, *P* ≤ 0.05 compared with OA (1 μM)-group (**B**–**D**).

It has been shown that intestinal TGR5 activation has the potential of inducing GLP-1 secretion via intracellular cAMP production [29]. To confirm this mechanism, we initially investigated the regulatory effect of a TGR5 agonist, OA, on in vitro GLP-1 secretion in enteroendocrine cells, the NCI-H716 endocrine-differentiated human colonic cell. The mRNA expression levels of *GPBAR1* encoding TGR5 in NCI-H716 cells was higher than in human skeletal muscle tissue and lower than in human spleen tissue fully recognized to express TGR5 (Figure 1A). Consistent with the mRNA expression patterns, TGR5 protein was detected in NCI-H716 cells (Figure 1B). Having demonstrated the definite expression of TGR5, we then evaluated the stimulatory activities of OA on GLP-1 secretion in NCI-H716 cells. It was observed that OA induced GLP-1 secretion in this cell line in a concentration dependent manner (1, 5, and 10 μM) (Figure 1C). Concomitantly with an increased GLP-1 secretion, OA also significantly augmented intracellular cAMP production. This finding indicates that OA could induce GLP-1 secretion via TGR5 activation (Figure 1D). Previous evidence has demonstrated that the proglucagon gene is the precursor gene of *GLP-1* and that GLP-1 amide is generated by post-translational processing of the proglucagon peptide with PC3 [30]. Therefore, we next assessed the mRNA levels of *GCG* (proglucagon gene) and *PCSK1* encoding PC3. Remarkably, we found that OA stimulation significantly upregulated the levels of both mRNAs (Figure 1E). These results suggest that OA stimulates both synthesis and secretion of GLP-1 in human enteroendocrine cells.

### 3.2. Oleanolic Acid and Anagliptin Effectively Collaborate to Exert an Antidiabetic Effect in Diabetic OLETF Rats

**Figure 2:**
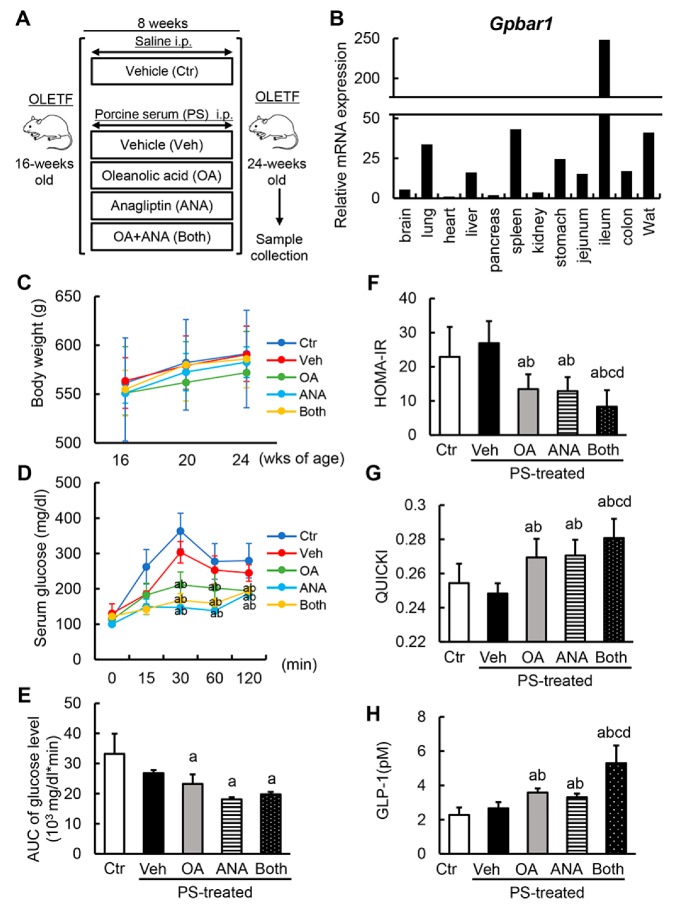
In vivo effects of oleanolic acid and anagliptin on glycemic status. (**A**) Schematic of porcine-serum (PS)-induced rat liver fibrosis models. (**B**) Histological distribution of *Gpbar1* mRNA expression in OLETF rats. Quantitative values are relatively indicated as ratios to the values of rat heart tissue. (**C**) Changes in body weights during experimental period. (**D**) Serum glucose levels in oral glucose tolerance test (OGTT) at the end of experiment. (**E**) The values of glucose–AUC (area under the blood concentration–time curve) in the experimental groups. The values of AUC were calculated as area under the curve of serum glucose levels at OGTT. (**F**,**G**) The values of homeostasis model assessment–insulin resistance (HOMA–IR) (**F**) and Quantitative Insulin Sensitivity Check Index (QUICKI) (**G**) in experimental rats. (**H**) The serum levels of rat GLP-1 at the end of experiment. Relative mRNA expression levels were measured by quantitative RT-PCR (qRT-PCR). *Gapdh* was used as internal control for qRT-PCR (**B**). Data are mean ± SD (**B**–**G**; *n* = 10, **H**; *n* = 8). Ctr; negative control group, Veh; vehicle-treated PS-injected group, OA; oleanolic acid-treated PS-injected group, ANA; anagliptin-treated PS-injected group, Both; oleanolic acid and anagliptin-treated PS-injected group. ^a^, *P* ≤ 0.05 compared with Ctr-group; ^b^, *P* ≤ 0.05 compared with Veh-group; ^c^, *P* ≤ 0.05 compared with OA-group; ^d^, *P* ≤ 0.05 compared with ANA-group.

Based on the stimulatory property of OA on GLP-1 synthesis and secretion, we evaluated the antidiabetic effect of OA in combination with ANA, a DPP-4 inhibitor. Experimental protocols are shown in Figure 2A. At the first onset, we confirmed a similarity of TGR5 expression’s histological distribution in diabetic OLETF rats and human organs. We observed a higher expression in the ileum, spleen, and white adipose tissue (Figure 2B). OLETF rats had a significantly higher body weight compared to nondiabetic LETO rats (Supplementary Materials Figure S1A). Additionally, the OLETF rats’ body weight remained unchanged following fibrotic induction with PS and administration of OA and/or ANA during the experimental period (Figure 2C). These results suggest that treatment with OA and ANA did not affect diabetic rats’ obesity.

Next, the alterations in the glycemic status following treatment with OA and ANA were assessed. At the experiment’s conclusion, an oral glucose tolerance test (OGTT) was performed to determine the differential glycemic status among the experimental groups. Based on the underlying characteristics, OLETF rats exhibited impaired glucose tolerance, and OA and/or ANA treatment significantly lowered serum glucose levels at 30, 60, and 120 min in OGTT (Supplementary Materials Figure S1B and Figure 2D). Estimation of AUC for plasma glucose in OGTT revealed that in the OLETF rats hyperglycemia significantly improved with both agents (Figure 2E). In keeping with glucose intolerance, OA and ANA treatment lowered the homeostasis model assessment of insulin resistance (HOMA–IR)’s high values in OLETF rats. Additionally, quantitative insulin sensitivity check index (QUICKI), a surrogate marker of insulin sensitivity, increased following the administration of both agents (Figure 2F,G). Of note, the combined administration of OA and ANA showed more potent improvement effects on both HOMA–IR and QUICKI vs the administration of either agent alone (Figure 2F,G). Next we aimed at determining whether treatment with OA stimulates GLP-1 secretion. For this, we evaluated serum GLP-1 levels. As expected, either OA or ANA-treated OLETF rats showed significantly higher levels of serum GLP-1 vs. vehicle-treated OLETF rats. Furthermore, interestingly, their combined administration further increased GLP-1 levels (Figure 2H).

### 3.3. Effects of Oleanolic Acid and Anagliptin on Hepatic Steatosis and Lipid Peroxidation in OLETF Rats

**Figure 3:**
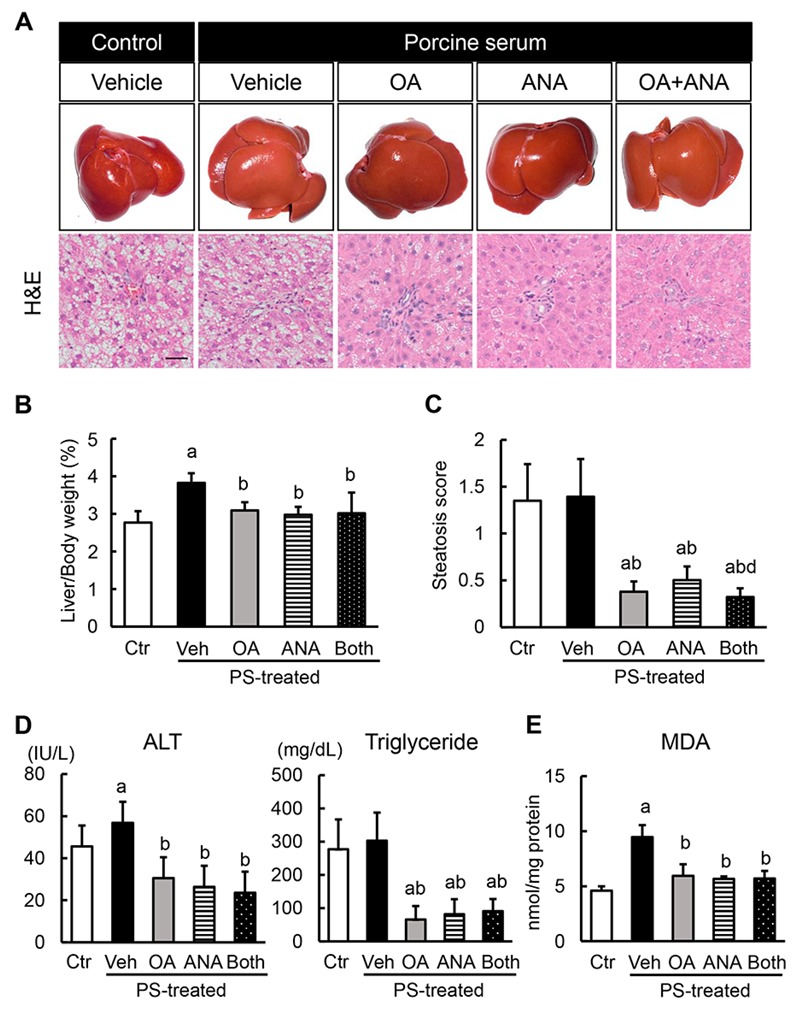
In vivo effects of oleanolic acid and anagliptin on hepatic steatosis and lipid peroxidation. (**A**) Representative macroscopic appearances (upper panels) and microphotographs of hematoxylin and eosin (HE) staining (lower panels) in the experimental groups. Scale bar; 50 μm. (**B**) Ratio of liver to body weight. (**C**) Histological score of steatosis according to NAFLD Activity Score. (**D**) Serum levels of alanine aminotransferase (ALT) and triglyceride (TG). (**E**) Hepatic concentrations of malondialdehyde (MDA). Data are mean ± SD (*n* = 10). Ctr; negative control group, Veh; vehicle-treated PS-injected group, OA; oleanolic acid-treated PS-injected group, ANA; anagliptin-treated PS-injected group, Both; oleanolic acid and anagliptin-treated PS-injected group. ^a^, *P* ≤ 0.05 compared with Ctr-group; ^b^, *P* ≤ 0.05 compared with Veh-group; ^c^, *P* ≤ 0.05 compared with OA-group; ^d^, *P* ≤ 0.05 compared with ANA-group.

Given the antidiabetic effects of OA and ANA, we analyzed the differential phenotypes in the liver of each experimental group. Despite the unchanged body weight, a fibrotic induction with PS increased the liver weight in OLETF rats. This increase was significantly attenuated by OA and ANA treatment (Figure 3A,B). Histological findings by hematoxylin and eosin staining revealed hepatic steatosis in OLETF rats, and hepatic steatosis in OLETF rats persisted without any changes under the condition of PS administration. Of note, OA and ANA treatment remarkably attenuated hepatic fat accumulation (Supplementary Materials Figure S1C and Figure 3A,C). In line with the altered histological features, higher alanine aminotransferase (ALT) and triglyceride serum levels were observed in the OLETF rats than in the LETO rats. Such elevated levels declined following treatment with both agents (Supplementary Materials Figure S1D and Figure 3D). It is known that reactive oxygen species play a key role both in inducing liver damage and initiating hepatic fibrogenesis [31]. Therefore, we next assessed the alterations in lipid peroxidation in the liver of the experimental groups. As for lipid accumulation, OLETF rats had elevated MDA hepatic levels, a marker of TBARS. This elevation was potently boosted by PS-mediated fibrotic induction (Supplementary Materials Figure S1E and Figure 3E). Consistent with attenuated hepatic steatosis, we found that the MDA levels were also significantly lowered by OA and ANA treatment (Figure 3E).

### 3.4. Effects of Oleanolic Acid and Anagliptin on Liver Fibrosis Development and Hepatic Stellate Cell Activation

**Figure 4:**
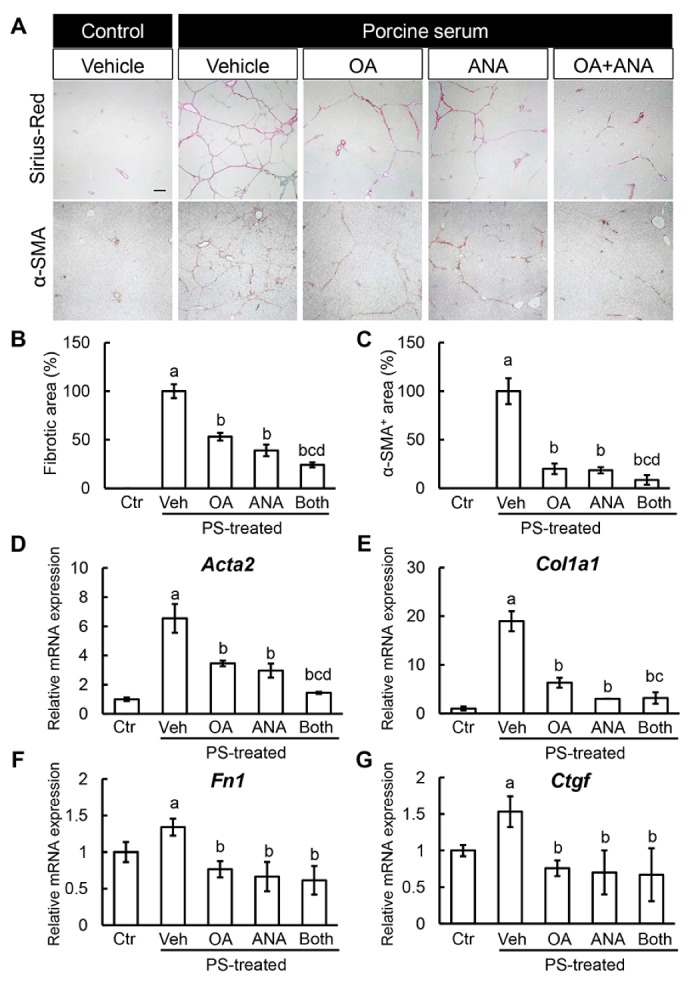
In vivo effects of oleanolic acid and anagliptin on liver fibrosis. (**A**) Representative microphotographs of liver sections stained with sirius red and α-SMA. Scale bar; 50 μm. (**B**,**C**) Semi-quantitation of sirius red-stained fibrotic area (B: Ctr; 0 ± 0%, Veh; 100.0 ± 7.0%, OA; 53.2 ± 3.9%, ANA; 39.1 ± 5.9%, Both; 24.2 ± 2.6%), and α-SMA immune-positive area (C: Ctr; 0 ± 0%, Veh; 100.0 ± 13.3%, OA; 20.1 ± 5.4%, ANA; 18.6 ± 3.1%, Both; 8.6 ± 5.0%) in high-power field by NIH imageJ software. (**D**–**G**) Relative mRNA expression levels of fibrosis markers, *Acta2* (D: Ctr; 1.00 ± 0.13, Veh; 6.54 ± 0.98, OA; 3.46 ± 0.19, ANA; 2.97 ± 0.48, Both; 1.44 ± 0.07), *Col1a1* (E: Ctr; 1.00 ± 0.41, Veh; 18.97 ± 2.06, OA; 6.30 ± 1.03, ANA; 3.02 ± 0.03, Both; 3.17 ± 1.16), *Fn1* (F: Ctr; 1.00 ± 0.14, Veh; 1.34 ± 0.12, OA; 0.77 ± 0.11, ANA; 0.66 ± 0.20, Both; 0.61 ± 0.19), and *Ctgf* (G: Ctr; 1.00 ± 0.07, Veh; 1.53 ± 0.21, OA; 0.76 ± 0.11, ANA; 0.70 ± 0.30, Both; 0.67 ± 0.36) in the liver of experimental mice. The mRNA expression levels were measured by quantitative RT-PCR (qRT-PCR), and *Gapdh* was used as internal control for qRT-PCR (D). Data are mean ± SD (*n* = 10). Ctr; negative control group, Veh; vehicle-treated PS-injected group, OA; oleanolic acid-treated PS-injected group, ANA; anagliptin-treated PS-injected group, Both; oleanolic acid and anagliptin-treated PS-injected group. ^a^, *P* ≤ 0.05 compared with Ctr-group; ^b^, *P* ≤ 0.05 compared with Veh-group; ^c^, *P* ≤ 0.05 compared with OA-group; ^d^, *P* ≤ 0.05 compared with ANA-group.

Based on the OA and ANA’s antisteatotic and antioxidant activities, we next evaluated the effects of both agents on liver fibrosis development. As shown in Figure 4A,B, 8-weeks of repeated PS administration sufficiently induced liver fibrosis development, which was indicated by complete formation of thin and straight septa between central veins as evaluated by sirius red stain in OLETF rats, and treatment with either OA or ANA improved PS-mediated liver fibrosis development. Of note, the combination of both agents drastically enhanced the antifibrotic effect of each single treatment. Thereafter, we performed an immunohistochemical analysis with α-SMA staining. The aim was to assess HSCs’ activation, which plays a crucial role in hepatic fibrogenesis. Remarkably, the combination treatment decreased the α-SMA positive areas in PS-mediated OLETF rats, along with liver fibrosis (Figure 4A,B). The observed ameliorations in the fibrotic phenotypes coincided with reduced hepatic expressions of profibrotic genes, including *Acta2*, *Col1a1*, *Fn1*, and *Ctgf* (Figure 4C).

**Figure 5:**
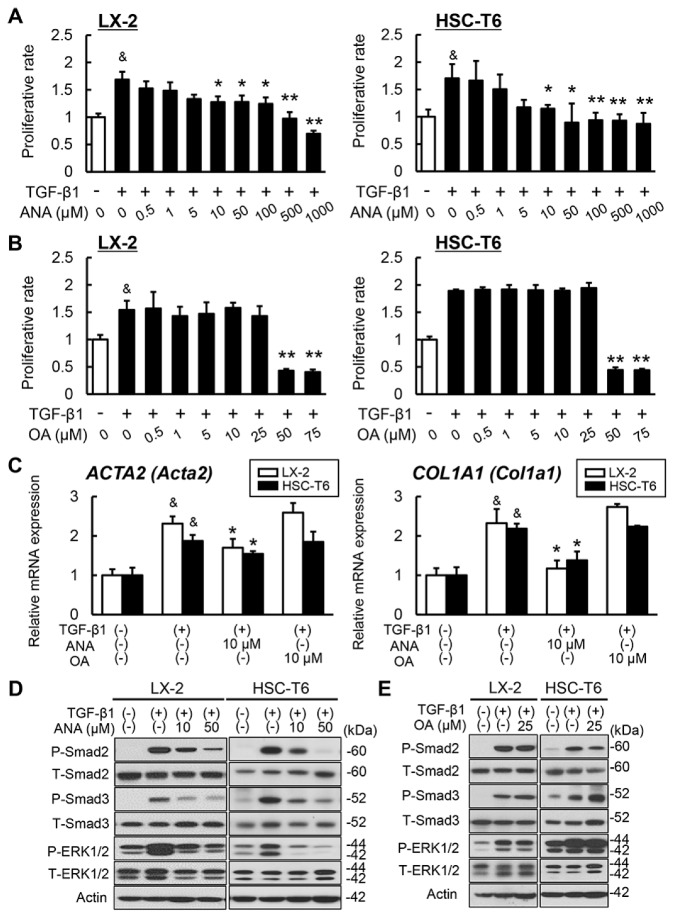
Effects of oleanolic acid and anagliptin in vitro hepatic stellate cells. (**A**,**B**) The effects of anagliptin (ANA) (**A**) or oleanolic acid (OA) (**B**) on the TGF-β1-stimulated proliferation of LX-2 and HSC-T6 cells. Both cell lines were cultured with different concentrations of ANA or OA for 24 h. The proliferative rate is the ratio to control group cultured without TGF-β1 and ANA or OA. (**C**) The effects of anagliptin (ANA) or oleanolic acid (OA) on the mRNA expressions of *ACTA2* (*Acta2*) and *COL1A1* (*Col1a1*) in the TGF-β-stimulated LX-2 and HSC-T6 cells. Both cell lines were cultured with 10 μM of ANA or OA for 24 h. Quantitative values are relatively indicated as ratios to the values of ANA(–)/OA(–)-group. The mRNA expression levels were measured by quantitative RT-PCR (qRT-PCR), and *Gapdh* was used as internal control for qRT-PCR. (**D**,**E**) Western blots of whole cell lysates from LX-2 and HSC-T6 for the phosphorylation of Smad2, Smad3, and ERK1/2. The cells were cultured with and/or without TGF-β and ANA (**D**) or OA (**E**). Actin was used as internal control for western blotting. Data are mean ± SEM (*n* = 8). ^&^, *P* ≤ 0.01 compared with TGF-β1(–)/ANA(–)/OA(–)-group; *, *P* ≤ 0.05; **, *P* ≤ 0.01 compared with TGF-β1(+)/ANA(–)/OA(–)-group.

We then planned to further explore the molecular mechanism underlying these antifibrotic properties. To this end, we aimed at revealing the direct effect of OA and ANA on in vitro LX-2 and HSC-T6 cells, human and rat Ac-HSCs, respectively. Cell proliferation assays demonstrated that ANA attenuated the proliferation of LX-2 and HSC-T6 cells upon TGF-β1 challenge in a dose-dependent manner (Figure 5A). In contrast, we observed that OA had no effect on the proliferation of these lines, and it induced toxic cell death at a concentration higher than 25 μM, in line with the absence of *GPBAR1* expression in the activated HSCs (Figure 1A,B and Figure 5B). Moreover, we found that the profibrogenic markers *Acta2* and *Col1a1*’s expression in both types of Ac-HSCs were profoundly decreased following the treatment with ANA while not with OA (Figure 5C). Of note, consistent with changes in the proliferative and profibrogenic capacity, treatment with ANA in both LX-2 and HSC-T6 cells suppressed TGF-β1-mediated phosphorylation of ERK1/2 and Smad 2/3 (Figure 5DMcKenzie, C.A.; Tirona, R.G.; Summers, K.; Seney, S.; Chakrabarti, S.; Malhotra, N.; Beaton, M.D. Sitagliptin in patients with non-alcoholic steatohepatitis: A randomized, placebo-controlled trial. World J. Gastroenterol. 2017, 23, 141–150.). Meanwhile, treatment with OA did not change these signal transductions in both lines (Figure 5E).

### 3.5. Changes in Fecal Microbial Profiles by Treatment with Oleanolic Acid and Anagliptin in OLETF Rats

**Figure 6:**
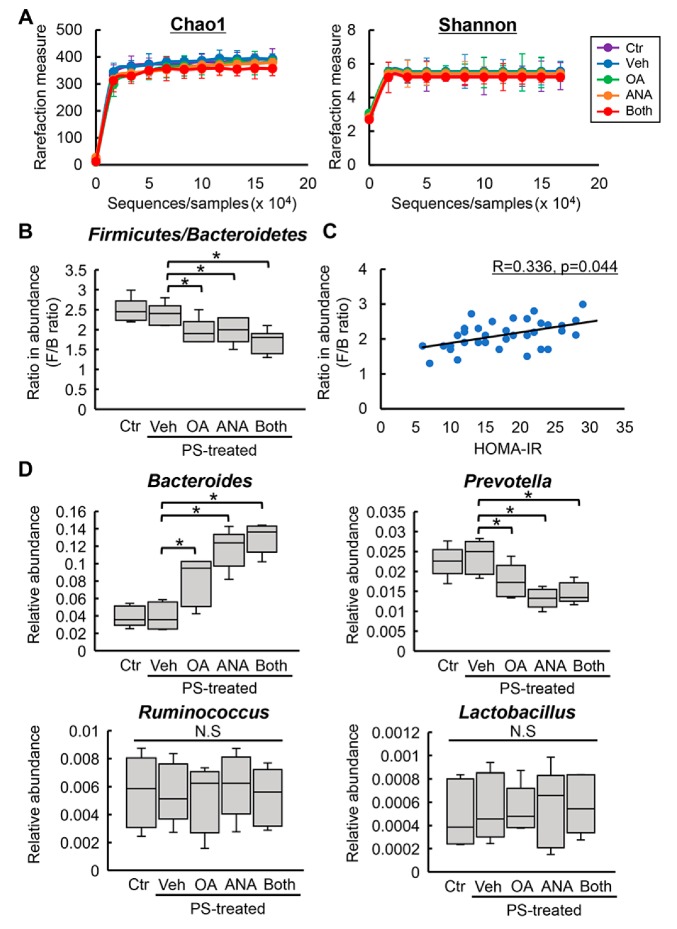
Changes in rat fecal microbiota by oleanolic acid and anagliptin. (**A**) Comparative analysis of Chao1 richness and Shannon diversity in fecal microbiome of the experimental groups. (**B**) The ratio of Firmicutes:Bacteroidetes (F/B) at the phylum level in the experimental groups. (**C**) Univariate correlation analysis between the ratio of F/B and the values of HOMA–IR in the experimental OLETF rats. (**D**) Relative abundances of *Bacteroides*, *Prevotella*, *Ruminococcus*, and *Lactobacillus* at the genus level in the experimental group. Ctr; negative control group, Veh; vehicle-treated PS-injected group, OA; oleanolic acid-treated PS-injected group, ANA; anagliptin-treated PS-injected group, Both; oleanolic acid and anagliptin-treated PS-injected group. Data are means ± SD (*n* = 5). *, *P* ≤ 0.05.

Recent reports have shown that the gut microbiota was modified by administration of a DPP4 inhibitor and TGR5’s activation [32,33]. Therefore, we next assessed fecal microbial profiles. A global structural analysis revealed no significant differences in the microbial richness and diversity, indicated by Chao and Shannon indexes, respectively, for the experimental groups (Supplementary Materials Figure S2A and Figure 6A). In short, compared to the normal state, the microbiota in the diabetic status features a higher percentage of Firmicutes and a lower percentage of Bacteroidetes, i.e., a higher ratio of Firmicutes:Bacteroidetes (F/B) at the phylum level [34,35,36]. In line with these observations, we found that OLETF rats had a higher F/B ratio in average abundance compared with LETO rats (Supplementary Materials Figure S2B). Of note, F/B’s increased ratio was inhibited by OA and ANA treatment (Figure 6B). Additionally, univariate correlation analysis demonstrated that in all OLETF rats, the F/B ratio positively correlated with the value of HOMA–IR (*R* = 0.336, *p* = 0.044, Figure 6C). In particular, at the genus levels, a higher abundance in *Bacteroides* and a lower abundance in *Prevotella* were observed in the feces of OLETF rats OA and/or ANA treated. On the contrary, the average abundance of *Ruminococcus* and *Lactobacillus* was not affected by treatment with both agents (Figure 6D).

## 4. Discussion

**Figure 7:**
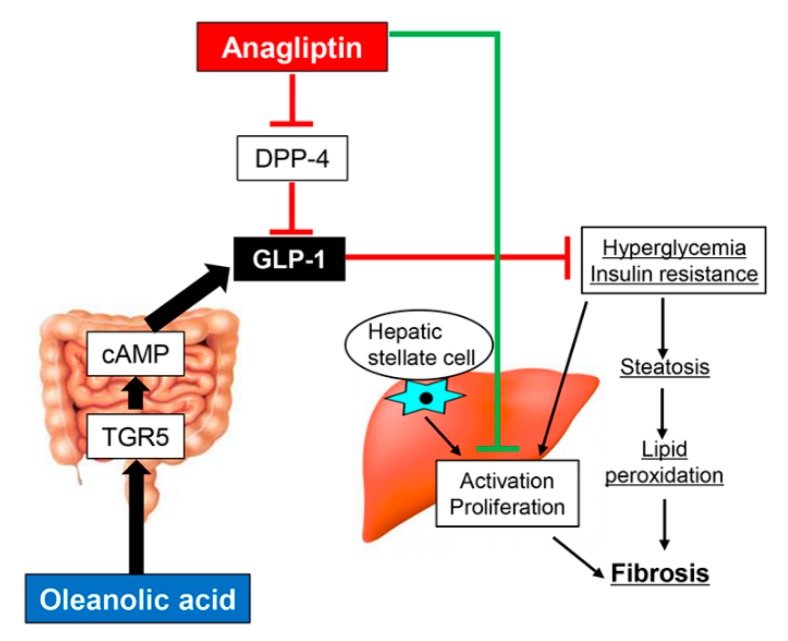
Schematic of antifibrotic effects of oleanolic acid and anagliptin.

The aim of this study was to explore a novel therapeutic strategy for diabetes-related liver fibrosis. To this end, we proposed a combined treatment with the DPP-4 inhibitor ANA and the TGR5 agonist OA. Our results demonstrated that in diabetic OLETF rats, the TGR5 agonist OA efficiently potentiated the antifibrotic activity of ANA against PS-induced hepatic fibrogenesis. We believe that this ANA-mediated antifibrotic effect is due to several underlying mechanisms (Figure 7). The first is based on its antidiabetic action. Our study demonstrates that ANA treatment suppressed hepatic steatosis. In turn, this ensues as a result of lipid peroxidation’s accumulation by improving hyperglycemia and IR. As a consequence, OLETF rats experience the development of an attenuated liver fibrosis. These findings are in line with earlier studies showing in NASH rodent models that sitagliptin, another DPP-4 inhibitor, inhibited liver fibrosis by suppressing steatosis and oxidative stress [37,38,39]. Additionally, a previous report from our group has found that activated HSCs’ proliferative potential was boosted under the co-existence of hyperglycemia and hyperinsulinemia at similar levels in OLETF rats [9]. An additional cell-based experiment has also found that high glucose stimulated proliferation via the NADPH oxidase-mediated reactive oxygen species, a mechanism recognized to be involved in HSC activation [40]. We believe that these evidences suggest that ANA possibly inhibited HSCs activation and proliferation by improving the glycemic status. Strikingly, we observed that OA-stimulated intestinal TGR5 activation had an inhibitory effect on liver fibrosis with antidiabetic and antioxidant properties even on its own. Additionally, it determined a pronounced augmentation of ANA-mediated effects.

Secondly, our in vitro assays found that ANA suppressed activated HSCs’ TGF-β1-stimulated proliferation and profibrogenic activity in normal conditions. Such findings suggest that HSCs were affected by ANA independently of altered glycemic status. It has been shown that in the liver, DPP-4 plays a role in fibronectin-mediated interaction of hepatocytes with the extracellular matrix. Furthermore, DPP-4 is expressed on the surface of reactive fibroblasts, including activated HSCs [41]. Wang et al. have shown that DPP-4 exhibits profibrotic behavior in carbon tetrachloride-induced liver fibrosis [42]. Based on these findings, several DPP-4 inhibitors (i.e., sitagliptin and alogliptin) have been reported to exert direct inhibitory effects on liver fibrosis development. Additionally, they play a role in suppression of proliferation and collagen synthesis in activated HSCs [11,43]. Similarly, we found that ANA directly relieved HSC activation by suppressing the phosphorylation of ERK1/2 and Smad 2/3. Unlike the effects of ANA, OA did not show a direct influence on the activated HSCs due to the absence of TGR5 expression. On the contrary, it was recently reported that OA-derivatives inhibit the proliferation and induce apoptosis of HSC-T6 cells [44]. However, the molecular mechanisms underlying these phenomena remain unclear. As a consequence, further investigation is necessary to clarify this discrepancy and the process at its basis.

In the present study, we also assessed fecal microbial alterations by ANA and OA. The results of our investigation found that in OLETF rats, ANA normalized the increased phylum F/B ratio, a well-known dysbiosis indicator related to metabolic syndromes [34,35,36]. A recent study on a rat diabetic model has found that vildagliptin treatment also provided a similar microbial modification following a high-fat diet/streptozotocin injection [32]. Interestingly, we identified a normalization of this F/B ratio in the OA-treated group. This effect is in line with a report showing that GLP-1 therapy can enrich Bacteroidetes in streptozotocin-induced diabetic rats [45]. By contrast, earlier rodent research associated with dysbiosis suggested that OA reduced the abundance of the phylum Bacteroidetes in parenteral nutrition-treated rats [33]. These conflicting findings may be linked to the susceptibility of Bacteroidetes’s gut enrichment based on the nutritional status. Moreover, we observed marked changes in the dominant genus within the Bacteroidetes. Such results indicate a decreased abundance of *Prevotella* following ANA and OA treatment. These findings seem to play a role in diabetic status improvement, in line with a recent report showing that the population of *Prevotella* declined in OLETF rats following metformin exposure [46]. Meanwhile, in contrast to earlier investigations, *Ruminococcus* and *Lactobacillus*, the genera belonging to Firmicutes, showed no changes in our study [32,46]. Since the reason for this discrepancy remains obscure to date, in the future we would like to pursue this line of research.

Several limitations can be identified in the present study. First, we focused in particular on OA’s effects as a TGR5 agonist on glucose metabolism. However, OA has been reported to show hepatoprotective effects with other molecular mechanisms. For example, Reisman et al. demonstrated that OA can activate the nuclear factor erythroid-2 related factor 2-Keap1 pathway [47]. Since this pathway also plays a key role in oxidative stress and HSCs activation, additional analysis of the relationship with OA-mediated antifibrotic effects in the present model is required. Second, the OA’s pharmacological impacts on the liver are controversially reported. Our results and those of other reports indicate hepatoprotective effects of OA treatment. However, it is imperative to note that some other studies have shown a detrimental effect following OA treatment. Liu et al. have disclosed that repeated administration of high OA doses alters bile acid homeostasis and induces cholestatic liver injury [48]. In our study, we observed that OA could exert antidiabetic and antifibrotic effects even at a lower dose compared to the minimal toxic dose reported in the previous study [49]. Additionally, neither high levels of serum alkaline phosphatase nor hyperbilirubinemia were observed in OA-treated OLETF rats (data not shown). However, future studies should address alterations of bile acid metabolism. Third, the present study evaluated the effects of both agents using only a single liver fibrotic model. The present PS-induced model has been shown to exhibit liver fibrosis formation without hepatic inflammation [50]. The main purpose of this study was to identify the efficacy of both agents on liver fibrosis but not inflammation. As a consequence, we believed it would be better to remove influences on hepatic inflammation. In order to further elucidate whether these affect hepatic inflammation in the process of NASH, additional investigation is required by using other experimental models.

Taken together, OA, a TGR5 agonist, appears to potentiate the inhibitory effects on liver fibrosis development mediated by ANA, a DPP-4 inhibitor, in diabetic rats. Importantly, the pharmacological actions of both agents were achieved at a pharmacological dose without adverse effect. Thus, we believe that given our experimental results, this combination regimen may represent a potential novel strategy for antifibrotic therapy against diabetes-based liver fibrosis.

## Supplementary Materials

The following are available online at https://www.mdpi.com/2073-4409/8/10/1153/s1, Figure S1: Phenotypical differences between LETO and OLETF rats, Figure S2: Differences in fecal microbiota between LETO and OLETF rats, Table S1: List of primers used in q-PCR, Table S1: List of primers used in q-PCR.

## Abbreviations

ALT	Alanine aminotransferase
ANA	Anagliptin
DMEM	Dulbecco’s modified Eagle’s medium
DPP4	Dipeptidyl peptidase-4
ERK	Extracellular signal-regulated kinase
FBS	Fetal bovine serum
GLP	Glucagon-like peptide
HOMA-IR	Homeostasis model assessment of insulin resistance
HSC	Hepatic stellate cell
IR	Insulin resistance
LETO	Long–Evans Tokushima Otsuka
MDA	Malondialdehyde
NAFLD	Non-alcoholic fatty liver disease
NASH	Non-alcoholic steatohepatitis
OA	Oleanolic acid
OGTT	Oral glucose tolerance test
OLETF	Otsuka Long–Evans Tokushima Fatty
PS	Porcine serum
QUICKI	Quantitative insulin sensitivity check index
TGR	Takeda G protein-coupled receptor
