# Supplementary material for: TGR5 Activation Modulates an Inhibitory Effect on Liver Fibrosis Development Mediated by Anagliptin in Diabetic Rats
Source: Cells. 2019 Sep 26;8(10):1153. doi: 10.3390/cells8101153 (PMC6829474; doi:10.3390/cells8101153)
Supplement: Supplementary file 1 [file cells-08-01153-s001.zip › Supplementary information/Supplementary Material.docx]

*Supporting information*

TGR5 Activation Modulates an Inhibitory Effect on Liver Fibrosis Development Mediated by Anagliptin in Diabetic Rats

Daisuke Kaya ^1^, Kosuke Kaji ^1,^*, Yuki Tsuji ^1^, Satoko Yamashita ^2^, Koh Kitagawa ^1^, Takahiro Ozutsumi ^1^, Yukihisa Fujinaga ^1^, Hiroaki Takaya ^1^, Hideto Kawaratani ^1^, Kei Moriya ^1^, Tadashi Namisaki ^1^, Takemi Akahane ^1^ and Hitoshi Yoshiji ^1^

^1^ Third Department of Internal Medicine, Nara Medical University, Kashihara, Nara 634-8521, Japan

^2^ Sanwa Kagaku Kenkyusho, Co., Ltd., Nagoya, Aichi 461-8631, Japan

***** Correspondence: kajik@naramed-u.ac.jp Phone: +81-744-22-3051

**1. Supplementary materials and methods**

Liver fibrosis is a common feature of chronic liver injuries caused by a variety of etiologies (e.g., hepatitis B, hepatitis C, autoimmune disorders, alcohol abuse, and non-alcoholic fatty liver disease (NAFLD)) [1–3]. Pathologically, liver fibrosis is characterized by hepatic stellate cell (HSC) activation and excessive accumulation in the extracellular matrix. The progression of liver fibrosis is often influenced by various pathological conditions. Among others, the diabetic conditions type 2 diabetes mellitus (T2DM) and insulin resistance (IR) are crucial to aggravate fibrogenesis [4,5]. Several epidemiological studies revealed that IR represents an advanced fibrosis risk factor in patients with chronic hepatitis C [6,7]. Of note, T2DM

***Estimation of glycemic status***

At the experiment’s conclusion, all of the rats in each group were orally administered 2 g/kg of glucose. The plasma glucose levels were then measured at 0, 15, 30, 60, and 120 min with the oral glucose tolerance test (OGTT). IR and insulin sensitivity were evaluated with the homeostasis model assessment of insulin resistance (HOMA-IR) and the quantitative insulin sensitivity check index (QUICKI), respectively, as described previously (1).

***Histological and immunohistochemical analyses***

Liver sections were fixed with 10% formalin and embedded in paraffin. Subsequently, 5-μm paraffin sections were stained with hematoxylin and eosin and Sirius Red. Histological score of steatosis according to NAFLD Activity Score (2). Primary antibodies against α-SMA (ab124964) (Abcam, Cambridge, UK) were used for immunostaining. Specifically, staining was performed as per supplier’s recommendations. NIH ImageJ software (http://imagej.nih.gov/ij/) was used to perform quantitative analysis.

***Analysis of fecal microbiota***

At the end of experiments, feces were obtained from the terminal ileum of five rats in each experimental group. In order to obtain microbiome DNA, feces were treated with NucleoSpin DNA Stool kit (Macherey-Nagel, Duren, Germany). The DNA samples were analyzed by Next Generation Sequencing at Takara Bio Inc. The V4 hypervariable region of the bacterial 16S rRNA gene was ampliﬁed from the fecal DNA extracts. To this end, modiﬁed universal bacterial primer pairs 341F (5′-TCGTCGGCAGCGTCAGATGTGTATAAGAGA CAGCCTACGGGNGGCWGCAG-3′) and 806R (5′-GTCTCGTGGGCTCGGA GATGTGTATAAGAGACAGGGACTACHVGGGTWTCTAAT-3′) with Illumina adaptor overhang sequences were used. Amplicons were generated, cleaned, indexed, and sequenced according to the Illumina MiSeq 16S Metagenomic Sequencing Library Preparation protocol (http://support.illumina.com/downloads/16s_metagenomic_sequencing_library_preparation.html).

Sequencing data were combined and sample identiﬁcation was assigned to multiplexed reads using the MOTHUR software environment (3). Subsequently, data were denoized and low-quality sequences, pyrosequencing errors, and chimeras were removed. Sequences were then clustered into operational taxonomic units (OTUs) at 97% identity using the CD-HITOTU pipeline (available from http://eeizhong-lab.ucsd.edu/cd-hit-otu) (4). Due to the likelihood of a sequencing artifact, OTUs containing fewer than four reads per individual diet/animal combination were excluded. The Ribosomal Database Project Classiﬁer was used to perform taxonomic classiﬁcation of OTUs (5).

**2. References**

1. Katz A, Nambi SS, Mather K, Baron AD, Follmann DA, Sullivan G, et al. Quantitative insulin sensitivity check index: a simple, accurate method for assessing insulin sensitivity in humans. J Clin Endocrinol Metab. 2000;85(7):2402-10.
2. Kleiner DE, Brunt EM, Van Natta M, Behling C, Contos MJ, Cummings OW, et al. Design and validation of a histological scoring system for nonalcoholic fatty liver disease. Hepatology. 2005;41(6):1313-21.
3. Schloss PD, Westcott SL, Ryabin T, Hall JR, Hartmann M, Hollister EB, et al. Introducing mothur: open-source, platform-independent, community-supported software for describing and comparing microbial communities. Appl Environ Microbiol. 2009;75(23):7537-41.
4. Li W, Fu L, Niu B, Wu S, Wooley J. Ultrafast clustering algorithms for metagenomic sequence analysis. Brief Bioinform. 2012;13(6):656-68.
5. Wang Q, Garrity GM, Tiedje JM, Cole JR. Naive Bayesian classifier for rapid assignment of rRNA sequences into the new bacterial taxonomy. Appl Environ Microbiol. 2007;73(16):5261-7.

**3. Supplementary figure legends**

**Supplementary Figure 1. Phenotypical differences between LETO and OLETF rats**

OLETF rats showed higher body weights (A), impaired glucose tolerance (B), hepatic steatosis (C), elevated serum levels of alanine aminotransferase (ALT) and triglyceride (D) and increased hepatic accumulation of malondialdehyde (**MDA) (E).**

Data are mean ± SD (n = 10). Scale bar; 50μm. *,*P* ≤ 0.05; **,*P* ≤ 0.01 compared with LETO rats.

**Supplementary Figure 2. Differences in fecal microbiota between LETO and OLETF rats.**

(A) Comparative analysis of Chao1 richness and Shannon diversity in fecal microbiome of LETO and OLETF rats. (B) The ratio of *Firmicutes*: *Bacteroidetes* (F/B) at the phylum level in LETO and OLETF rats.

Data are mean ± SD (n = 5). **,*P* ≤ 0.01 compared with LETO rats.

**4. Supplementary Table1. List of primers used in q-PCR**

| **Gene** | **Sense (5’-3’)** | **Antisense (5’-3’)** |
| --- | --- | --- |
| **Rat** | | |
| **Gpbar1** | **GAGGGGTTCAGGAGCTTTCC** | **CAGATTGGCAAGCAGGGAGA** |
| **Acta2** | **ACTGGGACGACATGGAAAAG** | **CATCTCCAGAGTCCAGCACA** |
| **Col1a1** | **TGCTGCCTTTTCTGTTCCTT** | **AAGGTGCTGGGTAGGGAAGT** |
| **Fn1** | **TGTCACCCACCACCTTGA** | **CTGATTGTTCTTCAGTGCGA** |
| **Ctgf** | **AAATAAACTGCCTCCCAAACCA** | **GAAATGGCTTGCTCAGGGTAAC** |
| **Gapdh** | **CCGTGTTCCTACCCCCAATG** | **CCTTTAGTGGGCCCTCGGC** |
| **Human** | | |
| **GPBAR1** | **CACTGTTGTCCCTCCTCTCC** | **ACACTGCTTTGGCTGCTTG** |
| **GCG** | **GCACATTCACCAGCGACTACA** | **TGACGTTTGGCAATGTTGTTC** |
| **PCSK1** | **CAGAAGGCTTTTGAATATGGTGT** | **GGAGGCACTGCTGATGGAGAT** |
| **ACTA2** | **GAGACCCTGTTCCAGCCATC** | **TACATAGTGGTGCCCCCTGA** |
| **COL1A1** | **CCAAATCTGTCTCCCCAGAA** | **TCAAAAACGAAGGGGAGATG** |
| **GAPDH** | **CCAAGGAGTAAGACCCCTGG** | **TGGTTGAGCACAGGGTACTT** |
