# Supplementary figures and images for: TGR5 Activation Modulates an Inhibitory Effect on Liver Fibrosis Development Mediated by Anagliptin in Diabetic Rats
Source: Cells. 2019 Sep 26;8(10):1153. doi: 10.3390/cells8101153 (PMC6829474; doi:10.3390/cells8101153)

**Supplementary Figure 1**

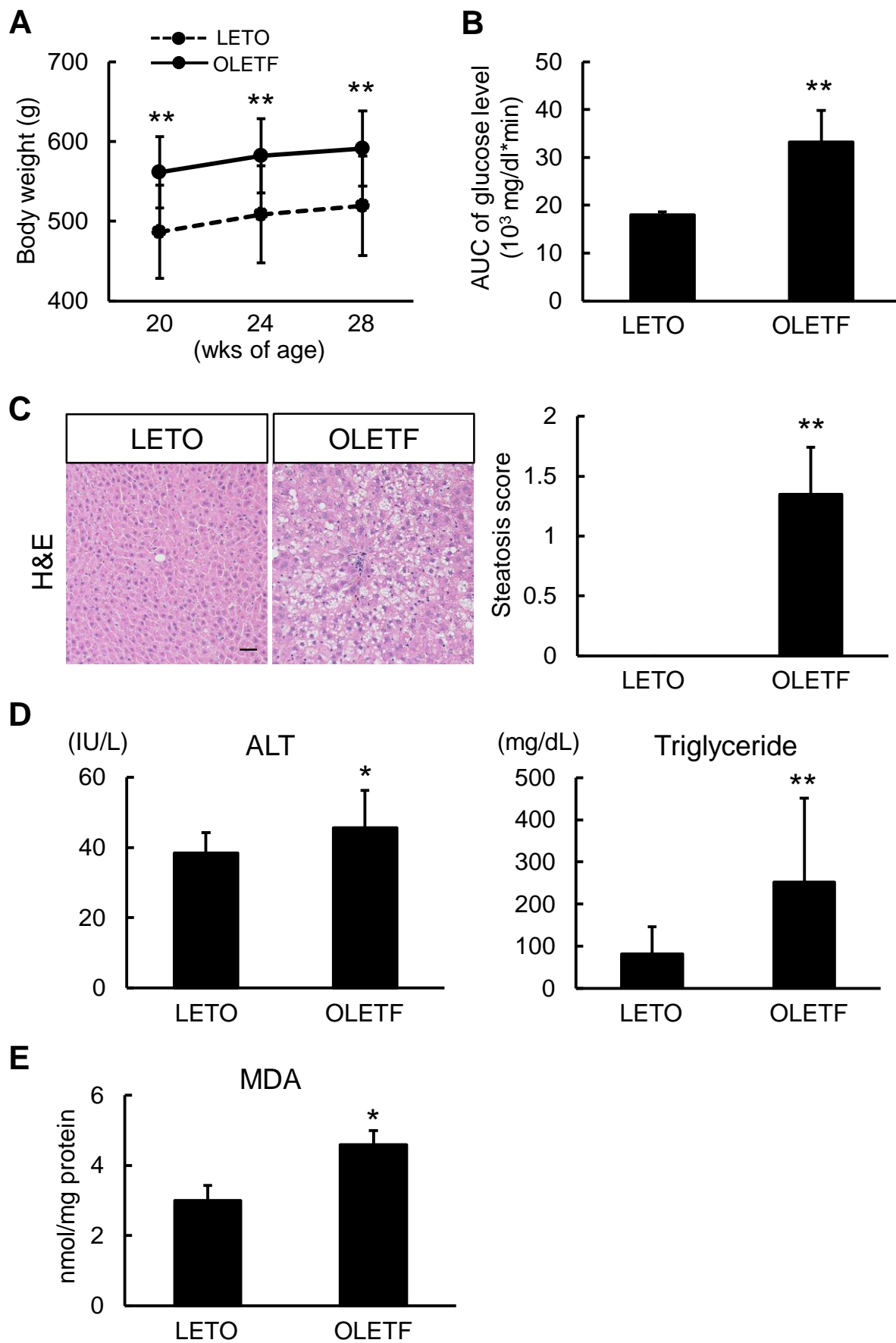

Supplement: Supplementary file 1 [file cells-08-01153-s001.zip › Supplementary information/Supplementary Figure 1.pdf]

## Supplementary Figure 2

**A**

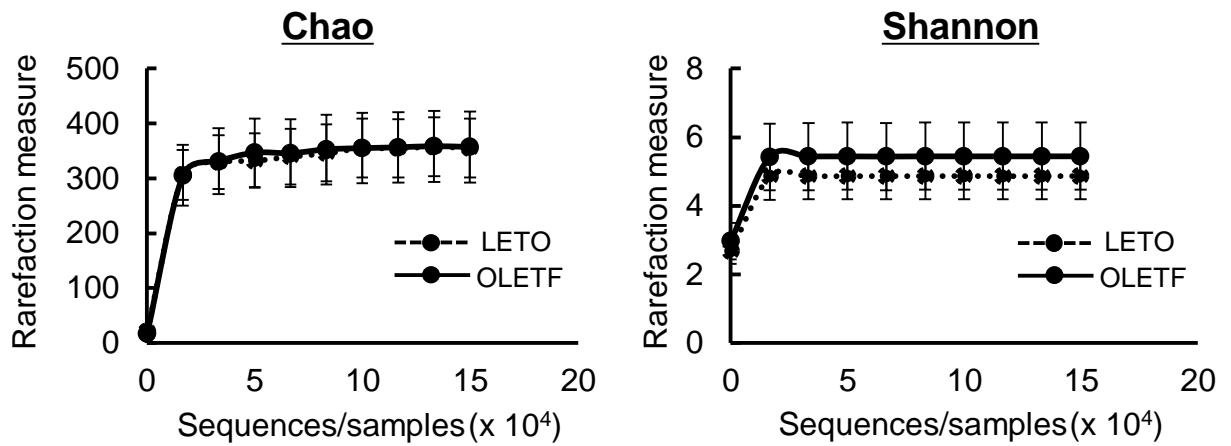

**B**

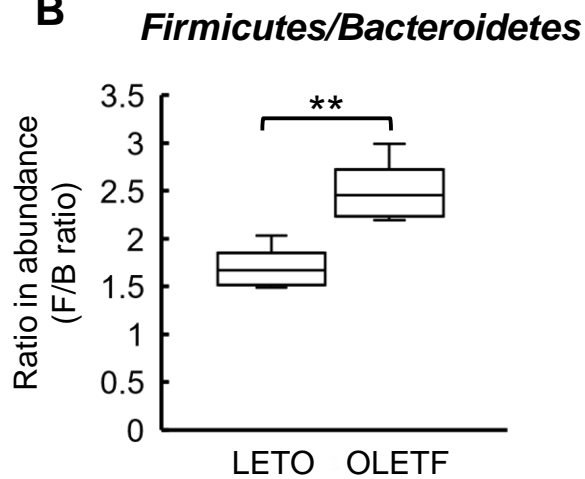

Supplement: Supplementary file 1 [file cells-08-01153-s001.zip › Supplementary information/Supplementary Figure 2.pdf]
